# Fog-based deep learning framework for real-time pandemic screening in smart cities from multi-site tomographies

**DOI:** 10.1186/s12880-024-01302-8

**Published:** 2024-05-27

**Authors:** Ibrahim Alrashdi

**Affiliations:** Department of Computer Science, College of Computer and Information Sciences, Jouf University, 72388 Sakaka, Aljouf Saudi Arabia

**Keywords:** Smart Cities, Pandemic diseases, Fog computing, Deep learning, Internet of Things (IoT)

## Abstract

The quick proliferation of pandemic diseases has been imposing many concerns on the international health infrastructure. To combat pandemic diseases in smart cities, Artificial Intelligence of Things (AIoT) technology, based on the integration of artificial intelligence (AI) with the Internet of Things (IoT), is commonly used to promote efficient control and diagnosis during the outbreak, thereby minimizing possible losses. However, the presence of multi-source institutional data remains one of the major challenges hindering the practical usage of AIoT solutions for pandemic disease diagnosis. This paper presents a novel framework that utilizes multi-site data fusion to boost the accurateness of pandemic disease diagnosis. In particular, we focus on a case study of COVID-19 lesion segmentation, a crucial task for understanding disease progression and optimizing treatment strategies. In this study, we propose a novel multi-decoder segmentation network for efficient segmentation of infections from cross-domain CT scans in smart cities. The multi-decoder segmentation network leverages data from heterogeneous domains and utilizes strong learning representations to accurately segment infections. Performance evaluation of the multi-decoder segmentation network was conducted on three publicly accessible datasets, demonstrating robust results with an average dice score of 89.9% and an average surface dice of 86.87%. To address scalability and latency issues associated with centralized cloud systems, fog computing (FC) emerges as a viable solution. FC brings resources closer to the operator, offering low latency and energy-efficient data management and processing. In this context, we propose a unique FC technique called PANDFOG to deploy the multi-decoder segmentation network on edge nodes for practical and clinical applications of automated COVID-19 pneumonia analysis. The results of this study highlight the efficacy of the multi-decoder segmentation network in accurately segmenting infections from cross-domain CT scans. Moreover, the proposed PANDFOG system demonstrates the practical deployment of the multi-decoder segmentation network on edge nodes, providing real-time access to COVID-19 segmentation findings for improved patient monitoring and clinical decision-making.

## Introduction

Pandemic diseases have become increasingly confronting for public infrastructure globally, with their extensive transmission and severe effects on individuals and communities. The rapid and perfect diagnosis of these diseases is of paramount importance for effective control and mitigation strategies [1]. The landscape of healthcare technology has been encountering a revolutionary shift in the wake of the COVID-19 pandemic, which highlighted the serious need for improved and adaptive solutions that can provide rapid and accurate diagnosis of pandemic diseases, particularly in urban environments where population density and mobility amplify the challenges of pandemic management [2].

Smart cities, the epitome of urban innovation, demonstrate the revolutionary role of integrating technologies in urban management. Specifically, the recent challenges modeled by the COVID-19 pandemic have prompted the conjunction of smart city technologies and pandemic control mechanisms. The process of screening pandemic disease is an essential element of public health surveillance and is now being reimagined and sustained through the application of cutting-edge technologies including real-time data analytics, predictive analytics, and fast reply apparatuses are at the vanguard of this evolving method [2]. Leading nations in this new paradigm of pandemic containment in smart cities have been identified. For instance, Singapore has put in place a national contact tracing app that uses Bluetooth technology to find and notify anyone who might have come into contact with COVID-19 cases that have been confirmed. Efficient control of outbreaks has been made possible by South Korea's strong IT infrastructure, vigorous testing, and data-sharing attitude [3]. Furthermore, Taiwan's creative integration of medical records and travel history to identify possible cases was partly responsible for the pandemic's successful containment. These examples demonstrate how smart city technologies can redefine the parameters and extent of pandemic control.

As countries throughout the world struggled to contain the outbreak, smart cities and the incorporation of internet technologies showed promise as a way to improve healthcare delivery and reaction times [4, 5]. The necessity of utilizing data-rich surroundings to promote accurate illness diagnosis and proactive decision-making in urban settings is now more important than ever in the post-COVID era. Smart cities have developed as centers of innovation that harness new technologies to address public health concerns. Specifically, the Artificial Intelligence of Things (AIoT) technology has developed as the result of the convergence between artificial intelligence (AI) and the Internet of Things (IoT) to offer a new paradigm to control pandemic diseases based on the data distributed across different geographical locations [6, 7].

The adoption of the AIoT framework in smart cities represents a paradigm shift in the way public health challenges are addressed. The urban areas utilize the capabilities of networked devices and sensors integrated inside diverse urban infrastructures to gather huge quantities of data in real-time. This data encompasses diverse sources such as healthcare facilities, environmental sensors, wearable devices, and social media platforms [4]. With the rapid proliferation AIoT technologies in smart cities, we can efficiently process and analyze this multi-site data to gain comprehensive insights into disease dynamics, patterns, and impacts on different segments of the population. AIoT facilitates the development and deployment of advanced machine learning algorithms, which can detect patterns, predict disease outbreaks, and enable proactive interventions. The application of AIoT technologies within smart cities has the capacity to significantly improve the precision and effectiveness of pandemic disease diagnosis owing to their ability to improve public health surveillance, response strategies for pandemics, and healthcare delivery systems [5]. To achieve sustainable management of pandemic disease in smart cities with high populations, it is highly required to interpret the distribution and seriousness of infected cases, which necessitates obtaining data from different healthcare sites in smart cities.

Smart healthcare systems usually focus on collecting medical imaging datasets to be used to build AI solutions for pandemic diseases during outbreaks. This data is usually sourced from varied imaging modalities and acquired from different healthcare institutions in the same smart city or even different cities, leading to inherent variability and heterogeneity across domains of data [6]. This cross-domain/multi-site bias arises as a possible consequence of variability in the specifications of equipment, scanning protocols, patient demographics, disease manifestations, and other facets. Indeed, variations in scanning details make significant inconsistencies in image quality, resolution, and noise levels, which complicate the process of feature extraction and interpretation [7]. In addition, the dependence on imagery data sourced from a single site or imaging modality may lead the model to overfit explicit data distributions, impeding its capacity to generalize to unseen data and varied clinical situations. This inherent bias brings a major limit to the generalization performance of ML in healthcare systems [8–10]. Hence, overlooking this bias usually makes the ML model operate in a suboptimal way, thereby reducing the reliability of related diagnostic decisions, and making ML non-applicable in real-world clinical environments. To tackle the problems posed by cross-domain/multi-site bias, it is required to offer a concerted effort to make use of recent advances in AIoT techniques, such as fog computing, which enables distributed processing of medical imaging data at the network edge [11].

As an essential part of the AIoT system, Fog computing has been used in the design of smart cities’ services to address the scalability and latency concerns by bringing the computing resources closer to edge devices. With this computing paradigm, fog computing allows for localized processing, alleviates the need for distant data transmission, and minimizes the overall system latency, which significantly accelerates the data analysis, and cooperative decision-making in a broad range of smart city applications, such as traffic management, environmental monitoring, and public safety [11]. Moreover, the utilization of this technology serves to bolster data security and privacy measures by confining important information to the confines of the local network. Fog computing facilitates the efficient usage of resources, low-latency operations, and improved overall performance in smart cities by effectively managing the substantial amount of data created by IoT devices. This capability plays a crucial role in fully harnessing the potential of smart city deployments [12, 13].

Our objective in this paper lies in developing a reliable AIoT framework to empower the efficiency of diagnosis processes for pandemic disease based on multi-site data fusion. The proposed framework aims to integrate diagnostic data from multiple sources, to acquire a comprehensive recognition of disease diagnosis and severity. The proposed AIoT framework is exemplified through a case study aiming at COVID-19 lesion segmentation [14]. By utilizing COVID-19 lesion segmentation as a specific application, the framework demonstrates its effectiveness in analyzing cross-domain CT scans and efficiently identifying infections. This case study serves as a practical implementation and validation of the proposed framework, showcasing its ability to accurately segment COVID-19 lesions and provide valuable insights for automatic COVID-19 pneumonia analysis. In this study, we introduce a novel technique called a multi-decoder segmentation network, which aims to enhance lung infection segmentation specifically for COVID-19 cases. a multi-decoder segmentation network is a lightweight approach that addresses the challenges posed by heterogeneous multi-site CT scans. It achieves robust performance by incorporating domain-adaptive normalization layers, which effectively handle inter-source data heterogeneity [15, 16]. Then, we propose a novel learning strategy that leverages heterogeneous knowledge interaction to facilitate cooperative learning of semantic representations from CT images sourced from diverse data sources. The proposed framework intelligently leverages fog computing capabilities to integrate a diagnostic model of an edge fog cloud prevailing in real-world smart cities.

The left part of the study is arranged as follows. Section 2 presents a comprehensive literature review, to highlight the existing research on multi-site data fusion, AIoT, and pandemic disease diagnosis. Next, we present the case study focusing on COVID-19 lesion segmentation. Section 4 describes our proposed multi-site data fusion framework and the AIoT-based learning strategy for accurate pandemic disease diagnosis. Section 5 discusses the system design specifications. Section 6 analyzes and interprets the results, discussing the implications and potential applications of our framework. Finally, we conclude the paper by summarizing the key findings and contributions.

### Related studies

This section presents a review of related research studies to gain constructive insights regarding the history of pandemic disease management in smart cities, and the related technologies in this subject matter.

#### Pandemic screening approaches

The literature contains a bunch of studies that have been instrumental in exploring innovative approaches and technologies to enhance screening and early detection of infectious diseases within urban environments. Allam and Jones [14] explored the amalgamation between AI and global data-sharing standards to allow for active control of urban health, explicitly throughout the outbreak of COVID-19 infection in smart cities. Their perspective paper was written one month following the initiation of the outbreak to provide surveys of the pandemic from an urban viewpoint aiming to figure out the way in which smart city networks can enable improved standardization conventions for amplified data sharing in case of epidemics. Costa and Peixoto [15] review the literature approaches for tackling the challenges imposed by the COVID-19 pandemic in smart cities. They also studied the potential solutions and reviewed the latest approaches that can be used in complicated pandemic settings, explaining reasonable and engaging development directions for the construction of health-centric smart cities. Shorfuzzaman et al. [16] studied the responsibility of video surveillance in the sustainability actions taken to control the COVID-19 pandemic in smart cities, with a primary emphasis on monitoring social distancing and ensuring mask-wearing. The authors have engaged in a discussion regarding the possible advantages of widespread video surveillance in terms of promoting public safety, facilitating traceability endeavors, and simplifying active resource management. Moreover, Ngabo et al. [17] examined the applicability of ML techniques for management pandemic across different tasks (including early diagnosis, epidemic detection, disease progression, death rates, and resource allocation), where extensive datasets were processed by ML algorithms to generate valuable insights that promote reliable decisions-making during epidemics.

#### AI for pandemic control in smart cities

The academic literature covers a diverse array of studies and research endeavors focused on utilizing AI to efficiently control and alleviate the consequences of pandemics in urban settings. In [18], Carmichael et al. conducted a retrospective multicenter study that involved the utilization of ML models to train a cohort of patients who were hospitalized with chronic liver disease with the aim of predicting the need for invasive mechanical ventilation (IMV) within a 48-hour timeframe specifically in patients diagnosed with COVID-19. Patients with chronic lung disease (CLD) were identified by the utilization of diagnosis codes about bacterial pneumonia, viral pneumonia, influenza, nonspecific pneumonia, and acute respiratory distress syndrome (ARDS). The candidate ML regressors encompass demographic and clinical characteristics that have been previously linked to unfavorable outcomes concerning COVID-19. Their proposed solutions were constructed by integrating logistic regression as well as three ensemble tree-based methods, namely decision tree, AdaBoost, and XGBoost. The models underwent validation in COVID-19 patients who were admitted to hospitals within two distinct healthcare systems over the period of March 2020 to July 2020. Wismüller et al. [19] introduced a methodology aimed at detecting pulmonary embolism in the early stages, specifically in the context of the COVID-19 pandemic. This strategy involved the collection of data from multiple institutions across the nation. They conducted an analysis on a comprehensive dataset obtained from several healthcare institutions in order to investigate the incidence of pulmonary embolism, a severe consequence linked to COVID-19 that has the potential to be fatal. In [20], Hooper et al. have collected inclusive data from 135 autopsy estimates of COVID-19-positive dead persons, involving histological assessment, in which postmortem inspections were executed by 36 diagnosticians at 19 health institutions or forensic centers in Brazil as well as the United States. The collection of each multi-site autopsy data was conducted through online submission of the response to open-ended surveys. Kaissis et al. [21] proposed an AI framework that preserves privacy in the context of multi-source medical imaging analysis, which aimed to tackle the issue of cooperating on medical image analysis across many institutions, while simultaneously safeguarding the privacy of patient data. The methodology described by the authors employs federated learning and safe aggregation methods to effectively train deep learning models on decentralized datasets while protecting the confidentiality of patient information.

#### IoT for pandemic control in smart cities

This part of the subsection explores the novel advancements and endeavors focused on the IoT within the framework of intelligent urban areas, explaining the utilization of these technologies to observe, alleviate, and address the difficulties presented by the pandemic. By conducting an extensive examination of relevant scholarly sources, we investigate the various applications and methodologies that underscore the potential of IoT-enabled solutions in effectively addressing public health emergencies within urban settings. Herath et al. [22] explored developing an intelligent system to alleviate the influence of the COVID-19 pandemic through exploring the ability of IoT technologies to allow active monitoring of patients, and reaction procedures throughout public health emergencies in urban areas. Their proposed system was configured to instantaneously collect real-time data on different attributes, counting ecological circumstances, social communications, and healthcare assets. The impact of the pandemic on the current state of smart city development was investigated by Gade et al. [23], who examined many elements including technical developments, infrastructure requirements, and regulatory changes. The researchers employed predictive modeling approaches to anticipate forthcoming trends and potential obstacles in the advancement of smart cities following the pandemic. Yang et al. [24] investigated the role of smart city projects in providing effective control of the COVID-19 pandemic with a specific focus on Chinese cities, in which in-depth analysis is dedicated to interpreting the roles of various IoT technologies in controlling the spread of the virus. They quantitatively evaluated the influence of smart city measurements and actions on decreasing the rates of infection or death and also on the rate of recovery. Umair et al. [25] conducted a study to investigate the influence of the COVID-19 pandemic on the implementation of IoT technologies across various domains within smart cities. The researchers investigated the impact of the pandemic on the utilization of IoT solutions in several regions. The purpose was to examine how these solutions have been employed to tackle issues and enhance operational effectiveness in these areas. The study conducted by Shorfuzzaman et al. [16] investigated the potential applications of video surveillance systems in monitoring public areas, enforcing social distancing measures, and improving public safety in the context of the pandemic. This study examined the potential advantages and difficulties linked to the implementation of mass video monitoring in smart cities, taking into account factors such as privacy apprehensions, data management, and ethical deliberations. The results of their study made a valuable contribution to the ongoing discussion surrounding the incorporation of surveillance technologies in the context of smart cities.

## Case study

**Fig.1:**
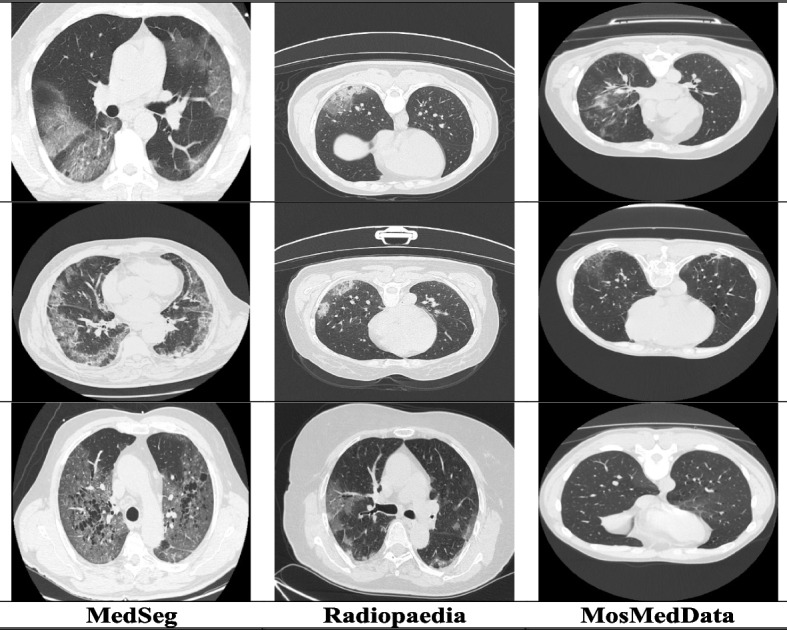
Visualization of the heterogeneity of COVID-19 CT samples orginating from different sites

To evaluate the effectiveness of the Multi-decoder segmentation network on multi-site CT data for COVID-19 diagnosis, we conducted experiments using three publicly accessible COVID-19 CT datasets. The first dataset [39], obtained from Radiopaedia, consisted of twenty COVID-19 CT volumes with over 1,800 annotated slices. The second dataset, known as MosMedData [40], comprised 50 CT volumes collected from public hospitals in Russia. Lastly, we utilized the MedSeg dataset [41], which included nine CT volumes containing a total of 829 slices, of which 373 were confirmed positive for COVID-19. Detailed information regarding the specific parameters of each dataset can be found in the corresponding research. Samples of the collected multi-site CT data are presented in Fig. 1. Following the preprocessing steps described in [12], we converted all three datasets into 2D images and applied random affine augmentation techniques to address any potential discrepancies. To ensure consistency across different facilities, we standardized the dimensions of all CT slices to 384 by 384, effectively reducing intensity variations. Prior to inputting the CT images into a multi-decoder segmentation network, we normalized their intensity scores to achieve a mean of zero and a variance of one. Our intensity normalization techniques encompassed bias field correction, noise filtering, and whitening, which were inspired by the methods outlined in [12] and have been validated for their effectiveness in optimizing heterogeneous learning. For our experimental setup, the multi-site data is split into 80% of the data allocated for training and the remaining 20% for testing. This partitioning allowed us to evaluate the performance of a multi-decoder segmentation network on unseen data and assess its generalization capabilities in the context of multi-site CT data fusion for COVID-19 diagnosis.

### Methodology

**Fig. 2:**
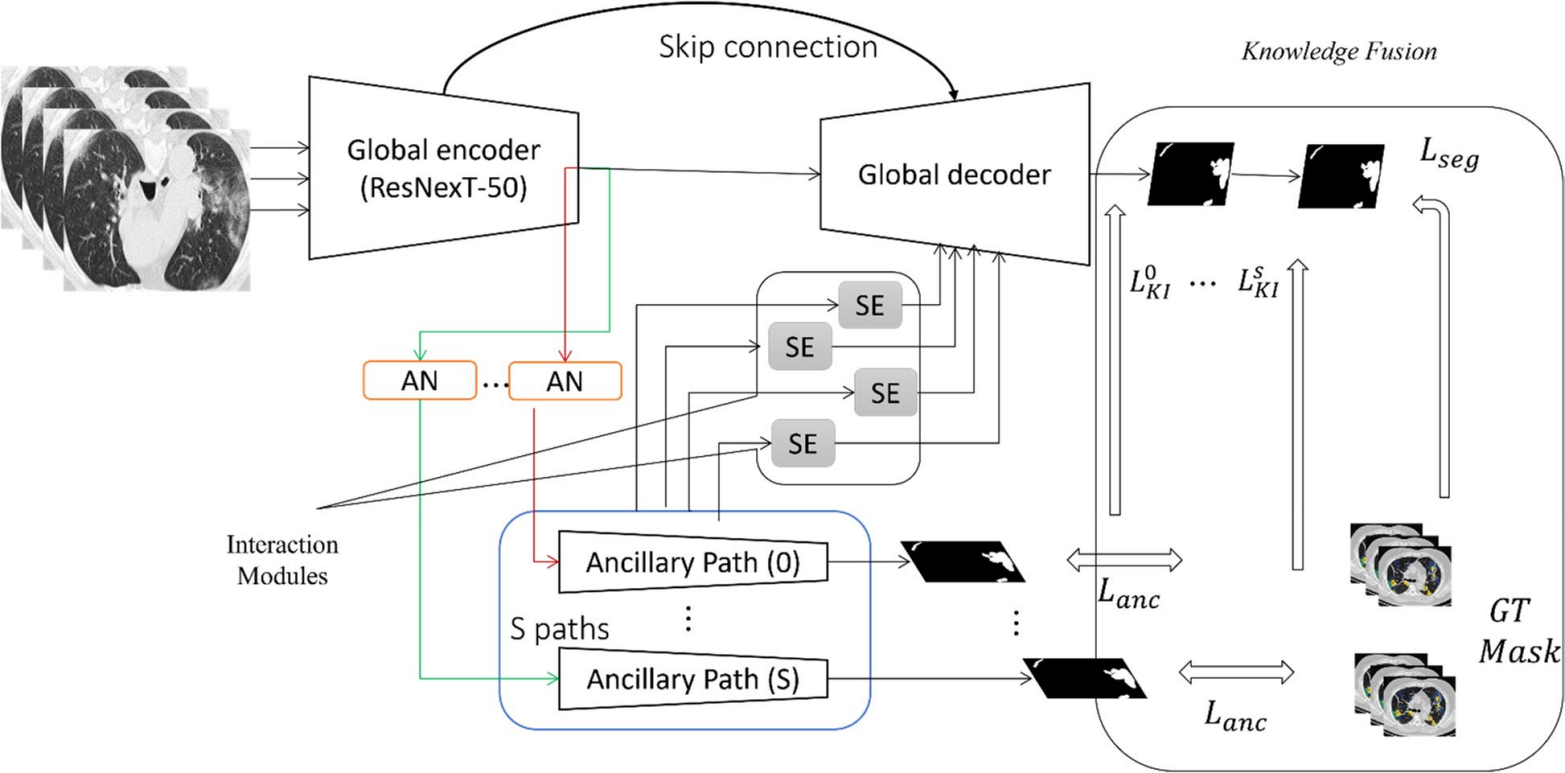
An illustrative diagram of the introduced multi-decoder segmentation network

In this section, we present the proposed multi-decoder segmentation network, which serves as the cornerstone of our study, specifically designed to address the segmentation of COVID-19 lesions in CT scans obtained from various sources within the context of pandemic diseases in smart cities. Figure. 2 provides a visual representation of the architecture.

Given the heterogeneity inherent in the data originating from different sources, our approach incorporates a comprehensive solution that includes both a global decoding path and dedicated ancillary decoding paths. This design allows the Multi-decoder segmentation network to effectively handle the challenges associated with segmenting COVID-19 lesions in CT scans from diverse origins. To tackle the inter-source variability, we introduce a re-parameterized normalization module within the ancillary decoding paths. This module plays a vital role in mitigating the impact of variations across different sources, enabling the multi-decoder segmentation network to adapt and generalize well to the unique characteristics of each dataset. By leveraging the learned heterogeneous knowledge from the ancillary paths, the ground truth (GT) masks contribute significantly to enhancing the overall network performance.

To further enhance the learning capability of the multi-decoder segmentation network, we incorporate interaction modules that facilitate the exchange of knowledge between the ancillary paths and the global paths at various levels within the network architecture. These interaction modules enable effective information sharing, enabling the multi-decoder segmentation network to leverage insights from different sources and improve the segmentation accuracy of COVID-19 lesions. This comprehensive approach ensures that the multi-decoder segmentation network can handle the specific challenges posed by the pandemic disease within smart city environments, leading to more accurate and reliable segmentation results.

#### Lesion encoder

Drawing inspiration from the architecture of U-Net [29], which comprises two convolutions and a max-pooling layer in each encoding block, we adopt a similar structure in a multi-decoder segmentation network. However, we enhance the feature encoding path by replacing the conventional encoder with a pre-trained ResNeXt-50 [30], retaining the initial four blocks and excluding the subsequent layers. Unlike traditional encoding modules, ResNeXt-50 incorporates a residual connection mechanism, mitigating the issue of gradient vanishing and promoting faster convergence during model training. Moreover, ResNeXt-50 employs a split-transform-merge strategy, facilitating the effective combination of multi-scale transformations. This strategy has been empirically shown to enhance the representational power of the deep learning model, particularly in capturing intricate features and patterns related to COVID-19 lesions in CT scans. By leveraging the strengths of ResNeXt-50 and its innovative architectural features, a multi-decoder segmentation network can effectively encode and extract relevant features from the input data. This enables the model to learn and represent complex spatial and contextual information, contributing to improved segmentation accuracy and the overall performance of our framework in addressing the challenges of COVID-19 lesion segmentation in smart city environments.

#### Domain-adaptive batch normalization layer

Recently, numerous medical imaging studies have adopted batch normalization (BN) [31] to alleviate interior covariate shift problems and to fine-tune the feature discrimination ability of CNNs; these accelerate the learning procedure. The key notion behind BN is to standardize the interior channel-wise representations, and subsequently perform an affine transformation on the generated feature maps that have optimizable parameters $\left[\gamma ,\beta \right]$. For specific channels ${x}_{k}\in \left[{x}_{1}\cdots \cdots ,{x}_{K}\right]$ In feature maps of K channels, their representations after being normalized ${y}_{k}\in \left[{y}_{1}\cdots \cdots ,{y}_{K}\right]$ are calculated as follows:

1 $${y}_{k}=\gamma .{\widehat{x}}_{k}+\beta , {\widehat{x}}_{k}=\frac{{x}_{k}-E\left[{x}_{k}\right]}{\sqrt{Var\left[{x}_{k}\right]}+\epsilon }$$

Symbols $E\left[x\right]$ and $Var\left[x\right]$ represent the average and variance of $x$, respectively, and $\epsilon$ represents an infinitesimal. The BN layer accumulates the flowing $E\left[x\right]$ and the flowing $Var\left[x\right]$ during training to learn the global representations and exploit these quantified values to normalize features at the testing stage.

In the context of smart cities, lung CT scans are sourced from diverse origins, utilizing different scanners and acquisition protocols. Figure 2 illustrates the statistics (mean and variance) obtained from individual data sources when training the deep learning models using a normalization layer exclusively for each source. The figure demonstrates notable variations in both mean and variance across different sources, particularly in intermediate layers where the feature channels are more abundant. These observed differences in statistics across heterogeneous sources present challenges when attempting to construct a unified dataset by combining all the diverse datasets. Firstly, the statistical disparities among the heterogeneous data can complicate the learning process of global representations, as the shared kernels may disrupt the domain-specific discrepancies that are irrelevant to the common features. Secondly, during model training, the BN layers might yield imprecise estimations of global statistics due to the presence of statistical variations from heterogeneous sources [42–44]. Consequently, directly sharing these approximate statistics during the testing stage is likely to result in a degradation in performance. Therefore, it becomes evident that a straightforward combination of all heterogeneous datasets is not beneficial in the context of smart cities. Instead, a more sophisticated approach is required to address the statistical discrepancies and leverage the unique characteristics of each data source, enabling the development of a robust and effective DL model for accurate segmentation of COVID-19 lesions in lung CT scans. To address these issues, a reparametrized version of the normalization module is integrated into the encoder network to normalize the statistical attribute of data from heterogeneous data sources. Every source $s$ has domain-relevant trainable parameters $\left[{\gamma }^{s},{\beta }^{s}\right]$. Given a specific channel ${x}_{k}\in \left[{x}_{1}\cdots \cdots ,{x}_{K}\right]$ from source $s$, the corresponding output ${y}_{k}^{s}$ is expressed in the form.

2 $${y}_{k}^{s}=\gamma .{\widehat{x}}_{k}^{s}+\beta , {\widehat{x}}_{k}^{s}=\frac{{x}_{k}-E\left[{x}_{k}^{s}\right]}{\sqrt{Var\left[{x}_{k}^{s}\right]}+\epsilon }$$

During the testing stage, our normalization layer applies the collected and accurate domain-relevant statistics used for the upcoming normalization of CT scans. Furthermore, we map these domain-relevant statistics to a shared latent space within the encoder, where membership to the source can be estimated through a mapping function denoted as $\phi ({x}_{i})$. This mapping function aligns the source-specific statistics with a shared, domain-agnostic representation space. As a result, our model can model the lesion features from different CT sources in a manner that is both source-aware and harmonized. This, in turn, makes the training process serve to harmonize discrepancies between data sources. This not only improves the capability to model domain-specific features but also promotes a more efficient and inclusive fusion of multi-site data, hence supporting the representational power of our framework.

#### Lesion decoder

The decoder module plays a crucial role in the gradual upsampling of feature maps, enabling the network to generate a high-resolution segmentation mask that corresponds to the original input image (refer to Fig. 2). It takes the low-resolution feature maps from the encoder and progressively increases their spatial dimensions while preserving the learned feature representations. In addition to upsampling, the decoder incorporates skip connections, establishing direct connections between corresponding layers in the encoder and decoder. By merging features from multiple resolutions, the decoder effectively utilizes both low-level and high-level features, allowing the network to capture contextual information at various scales.

Similar to the approach described in [29], we have implemented a powerful block to enhance the decoding process. The decoding path in our U-shaped model employs two commonly used layers: the upsampling layer and the deconvolution layer. The upsampling layer leverages linear interpolation to expand the dimensions of the image, while the deconvolution layer, also known as transposed convolution (TC), employs convolution operations to increase the image size. The TC layer enables the reconstruction of semantic features with more informative details, providing self-adaptive mapping. Hence, we propose the utilization of TC layers to restore high-level semantic features throughout the decoding path. Furthermore, to improve the computational efficiency of the model, we have replaced the traditional convolutional layers in the decoding path with separable convolutional layers. The decoding path primarily consists of a sequence of $1\times 1$ separable convolutions, $3\times 3$ separable TC layers, and $1\times 1$ convolutions, applied in consecutive order. This substitution with separable convolutions helps reduce the computational complexity while maintaining the effectiveness of the model.

#### Knowledge fusion(KF)

After addressing the disparities among data sources in smart cities, the subsequent objective is to leverage the heterogeneity of these sources to effectively learn fine-tuned feature representations. The essential purpose of the Knowledge Fusion (KF) module is to smooth the harmonious integration of various visual representations acquired from different sources of CT scans during the learning or encoding-decoding process [32]. As shown in Fig 2, these encoded representations usually include features from different domains of CT imaging and different spatial scales. The KF module plays a pivotal role in reducing the overall heterogeneity present in the multi-site data, allowing for a more coherent and comprehensive analysis. Through an interactive process, the KF is designed to allow seamless fusion of these representations, enhancing the overall robustness and informativeness of the integrated data. This fusion process aims to improve the model's ability to capture and leverage the varied characteristics and nuances within the data, ultimately contributing to more accurate and reliable disease diagnosis in smart cities [33, 34].

As depicted in Fig. 2, collaborative training is employed for the global network, combining supervision from GT masks and additional heterogeneous knowledge from ancillary paths. Specifically, each domain-specific ancillary channel is constructed in a manner identical to the global decoding path, resulting in a total of S domain-related ancillary channels within the global network. The ancillary paths serve as independent feature extractors for each supported data source in smart cities, allowing for a more inclusive fusion of relevant knowledge representations compared to the global decoding path. Each ancillary path is trained to optimize the dice loss [35]. Concurrently, the acquired heterogeneous knowledge representations from the ancillary paths are shared with the global network through an effective knowledge interaction mechanism. This enables the collective transmission of knowledge from all ancillary paths into a global decoding path, stimulating the common kernels in the global network to learn additional generic semantic representations. Accordingly, the final cost function for multi-decoder segmentation network training with data from source $s$ includes dice loss ${L}_{global}^{s}$ and a knowledge interaction loss ${L}_{KI}^{s}$.

Unlike present knowledge distillation approaches [36], our knowledge interaction loss associates the global probability maps (in the global network) with the GT masks from the ancillary path by transforming the GT masks into a one-hot design, preserving the dimensions reliability of the possibility maps. Thus, we denote the estimated one-hot label of an ancillary path as ${P}_{\text{anc}}^{s}\in {\mathbb{R}}^{b\times h\times w\times c}$. The activation values following the $softmax$ operation of global architecture are denoted as ${M}_{\text{global}}^{s}\in {\mathbb{R}}^{b\times h\times w\times c}$, with $b$ representing batch size, $h,$ and $w$ representing the height and width of feature maps, respectively, and $c$ representing the channel number. The knowledge interaction cost can be calculated by

3 $${L}_{KF}^{s} \left({P}_{anc}^{s}, {M}_{global}^{s}\right)=1-\frac{2\sum_{i}^{\varphi }{m}_{i}^{s}.{p}_{i}^{s}}{\sum_{i}^{\varphi }{\left({m}_{i}^{s}\right)}^{2}+\sum_{i}^{\varphi }{\left({p}_{i}^{s}\right)}^{2}}$$

where ${m}_{i}^{s}\in {M}_{global}^{s} and {p}_{i}^{s}\in {P}_{\text{anc}}^{s}$, and $\varphi$ represents the number of pixels in a single batch. The concept of *KF* stems from the proven advantages of large posterior entropy [32]. In our model, each ancillary path aims to effectively capture semantic knowledge from the underlying dataset by learning diverse representations and generating a wide range of predictions, thus providing a comprehensive set of heterogeneous information for the proposed multi-decoder segmentation network in smart cities. In supervised training, a multi-decoder segmentation network achieves rapid convergence when the capacity is very large. However, in *KF*, the global path needs to emulate both the GT mask and the predictions of multiple ancillary paths simultaneously. The proposed *KF* introduces additional heterogeneous (multi-domain) representation to standardize the multi-decoder segmentation network and increase its posterior entropy [32], enabling joint convolutions to leverage more powerful representations from different data sources. Moreover, the multi-path structure in *KF* may also contribute to beneficial feature regularization for the global encoding path by joint training with the ancillary paths, thereby enhancing the segmentation performance of the multi-decoder segmentation network.

The interaction modules play a crucial role in the proposed multi-decoder segmentation network, consisting of multiple interaction blocks that take the acquired knowledge representation from the ancillary paths and transfer it to the global path to enhance the overall segmentation performance. These modules need to be lightweight to avoid increasing the model's complexity. Additionally, the approach should improve gradient flow to accelerate training convergence while leveraging channel-wise relationships. To achieve this, we adopt the recently proposed squeeze and excitation (SE) technique [37], which recalibrates feature maps through channel-wise squeezing and spatial excitation (referred to as sSE), effectively highlighting relevant spatial positions.

Specifically, cSE squeezes the feature maps of the $U_{anc}\in\mathbb{R}^{W\times H\times} C^{'}$ ancillary paths along the channel dimension and perform spatial excitation on the corresponding feature map of the global network ${U}_{global}\in {\mathbb{R}}^{W\times H\times C}$, thereby transmitting the heterogeneous knowledge representation learned for fine-tuning the generalization capability of the model. $H\text{ and }W$ represent the dimensions of feature maps, and $C^{'}\text{ and }C$ represent the channel count corresponding to the feature maps in the ancillary path and global path, respectively. Herein, we deliberate a certain dividing policy to characterize the input tensor ${U}_{anc}=\left[{u}_{anc}^{\text{1,1}},{u}_{anc}^{\text{1,2}},\cdots \cdots ,{u}_{anc}^{i,j},\cdots \cdots ,{u}_{anc}^{H, W}\right]$, where ${U}_{anc}^{i,j}\in {\mathbb{R}}^{W\times H\times C^{'}}$ with $j\in \left\{\text{1,2},\cdots \cdots ,H\right\}$ and $i\in \left\{\text{1,2},\cdots \cdots ,W\right\}$. In the same way, the global path feature map ${U}_{global}=[{u}_{global}^{\text{1,1}},{u}_{global}^{\text{1,2}},\cdots \cdots ,{u}_{global}^{i,j},\cdots \cdots ,{u}_{global}^{H, W}$]. A convolution layer ($1\times 1$) is employed to execute spatial squeezing $\text{q }= {\text{W}}_{s}* {U}_{anc}$, where ${\text{W}}_s\in\mathbb{R}^{1\times1\times} C^{'}$, and producing projection map $\text{q}\in {\mathbb{R}}^{H\times W}$. This generated $q$ is fed into sigmoid function $\sigma \left(\cdot \right)$ to be rescaled into the range of [0,1], and the output is exploited for exciting ${U}_{global}$ spatially to generate ${\widehat{U}}_{global}=[\sigma ({q}_{\text{1,1}}){u}_{global}^{\text{1,1}},\cdots \cdots ,\sigma ({q}_{i,j}){u}_{global}^{i,j},\cdots \cdots ,{\sigma \left({q}_{H,W}\right)u}_{global}^{H, W}$].

#### Multi-decoder segmentation network Specifications and training

The proposed multi-decoder segmentation network is trained to optimize the objective function for upgrading the global encoder (${\uptheta }_{\text{e}}$), global decoder (${\uptheta }_{\text{d}}$), and ancillary paths (${\left\{{\theta }_{anc}\right\}}_{1}^{S}$). The objective function could be formulated according to

4 $${L}_{anc}=\sum_{s=1}^{S}{L}_{anc}^{s}+\eta \left({\Vert {\theta }_{e}\Vert }_{2}^{2}+\sum_{s=1}^{S}{\Vert {\theta }_{anc}^{s}\Vert }_{2}^{2}\right),$$

5 $${L}_{global}=\sum_{s=1}^{S}(\sigma { L}_{KI}^{s}+{\left(1-\sigma \right) L}_{global}^{s})+\eta \left({\Vert {\theta }_{e}\Vert }_{2}^{2}+{\Vert {\theta }_{d}\Vert }_{2}^{2}\right),$$

where ${\text{L}}_{\text{anc}}^{\text{s}}$ and ${L}_{\text{global}}^{s}$ represent the dice loss for the ancillary paths and the global path, respectively; ${L}_{KI}^{s}$ represents the knowledge interaction for the global network; $\sigma$ denotes a hyperparameter for balancing the segmentation loss and the knowledge interaction loss, and is set to 0.6, and $\eta$ denotes the weight parameter and is set to 0.0001.

Throughout the entire training process, knowledge interaction takes place. At each training step, S batches of CT scans, each belonging to a different dataset, are fed into the multi-decoder segmentation network. The ancillary paths and the global path are trained alternately. Once training is completed, the ancillary paths are removed, and only the global path remains for inference. The proposed multi-decoder segmentation network is implemented on NVIDIA Quadro GPUs, with one GPU assigned to each data source, using the TensorFlow library. The encoder is built with four ResNeXt blocks. We employ the Adam optimizer to update the parameters of the multi-decoder segmentation network. During training, a batch size of 5 is used, and the number of iterations is set to 25000.

### System design

**Fig. 3:**
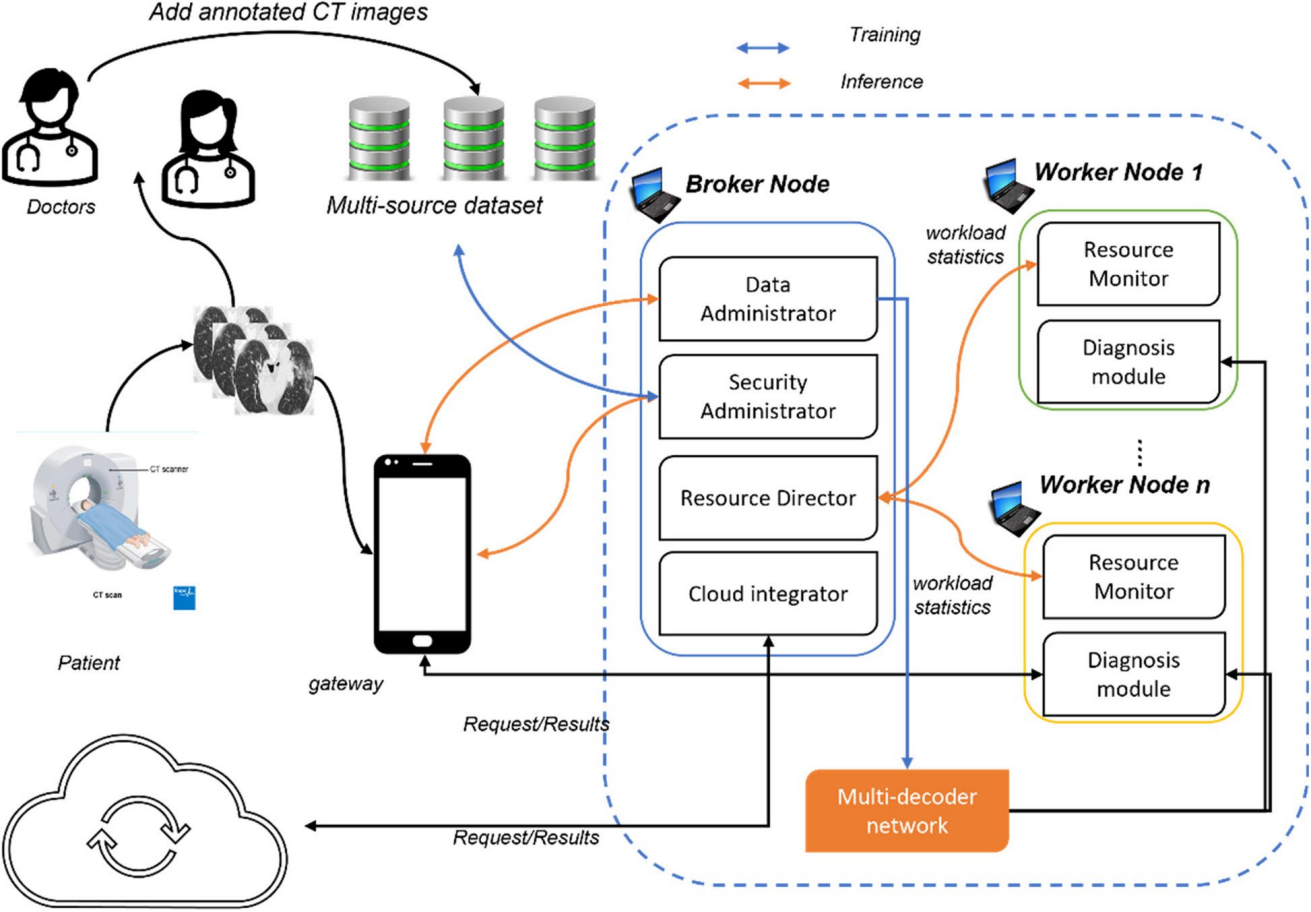
Systematic representation of the proposed PANDFOG framework

The suggested system in this work is a fog-empowered cloud computing framework for COVID-19 diagnosis in smart cities, known as PANDFOG. It utilizes the proposed multi-decoder segmentation network to segment infection regions from CT scans of patients, aiding doctors in diagnosis, disease monitoring, and severity assessment. PANDFOG integrates various hardware devices and software components to enable organized and unified incorporation of edge-fog-cloud, facilitating the rapid and precise transfer of segmentation outcomes. Figure. 3 provides a simple illustration of the PANDFOG architecture and its modules are discussed in the following subsections.

Gateway Devices: Smartphones, tablets, and laptops serve as gateways within the PANDFOG framework. These devices function as fog devices, aggregating CT scans from various sources and transmitting them to the broker or worker nodes for further processing. The broker node serves as the central reception point for segmentation requests, specifically CT images, originating from gateway devices. It comprises the request input component for handling incoming requests, the security administration component for ensuring secure communication and data integrity, and the adjudication component (resource director) for real-time workload analysis and allocation of segmentation requests [45–49].

The worker node is responsible for executing segmentation tasks assigned by the resource director. It includes embedded devices and simple computers such as laptops, PCs, or Raspberry Pis. Worker nodes in PANDFOG encompass the proposed multi-decoder segmentation network architectures for processing CT images from heterogeneous sources and generating segmentation results. Additional components for data preparation, processing, and storage are also integral parts of the worker node [51].

The software components of PANDFOG enable efficient and intelligent data processing and analysis, leveraging distributed computing resources at the network edge. These components collectively contribute to tacking the problems facing the screening of COVID-19 and thereby improving healthcare responses. The first computation in PANDFOG involves preprocessing CT scans before they are forwarded to the multi-decoder segmentation network for training or inference. Data preprocessing details are provided in the experimental part of the study. This module trains the proposed Multi-decoder segmentation network on heterogeneous CT images after the preparation phase. It utilizes the Multi-decoder segmentation network to infer segmentation outcomes for CT images received from gateway devices based on the resource director's assignment. The resource directory comprises the workload administrator and the adjudication component. The workload administrator manages segmentation requests and handles the request queue and a batch of CT images. The adjudication component regularly analyzes available cloud or fog resources to determine the most suitable nodes for processing CT scans and generating segmentation outcomes. This aids in load balancing and optimal performance [52–57].

The PANDFOG framework takes the patient's CT image as input from gateway devices and employs the data preparation module and Multi-decoder segmentation network to generate segmentation results indicating the infection regions. The Multi-decoder segmentation network is trained on multi-source annotated datasets and saved on all nodes. During the diagnosis phase, a node assigned with a segmentation request feeds the patient's CT image to the Multi-decoder segmentation network for forward pass inference. The input image is broadcast to other nodes if needed.

### Experimental deign and analysis

Within this section, we provide a comprehensive comparison between the outcomes achieved by our model and those reported in previous studies. Furthermore, we undertake two distinct evaluations to assess the performance and efficacy of our proposed multi-decoder segmentation network. The initial evaluation takes place within a conventional computing environment, allowing us to gauge the model's overall performance and effectiveness. Subsequently, we delve into a detailed analysis of the experimental configurations of the multi-decoder segmentation network within an AIoT framework, considering various factors such as latency, jittering, completion time, and more. This multifaceted evaluation provides a comprehensive understanding of the multi-decoder segmentation network's capabilities and performance within the context of AIoT, offering insights into its potential for practical applications [56, 57].

#### Performance *indicator*

In our case study, we employed two commonly used evaluation indicators to assess the performance of the multi-decoder segmentation network framework for COVID-19 lesion segmentation: the Dice Similarity Coefficient (DSC) and the Normalized Surface Dice (NSD).

6 $$DSC=\frac{2\left|S\cap G\right|}{\left|S\right|+\left|G\right|}$$

7 $$NSD=\frac{2\left|\partial S\cap {B}_{\partial S}^{\tau }\right|+2\left|\partial S\cap {B}_{\partial G}^{\tau }\right|}{\left|\partial S\right|+\left|\partial G\right|}$$

## Results and discussion

In our experiments, we explore and evaluate the proposed network under two training scenarios: one is an individualistic scenario and the other is a combined scenario. The former scenario emphasizes training the network separately on each dataset and then reporting its inference performance. In a later scenario, we involve the direct integration of arbitrarily selected images from different sources. This random selection of samples is performed to guarantee that the total number of training samples and test samples are equals in both scenarios. This in turn helps keep the fairness of the conducted comparisons. For both scenarios, the segmentation performance is assessed on test data from distinct sites, which helps gain useful insights about the generalizability of the model on unseen data. This means interpreting the impact of distributional shifts on the segmentation performance when dealing with multi-site CT data. Throughout these bunch of experiments, the multi-decoder segmentation network utilized a global decoding path, which is typical for U-shaped segmentation networks. The quantitative results of these experiments are reported in Table 1, across different valuation metrics namely DSC and NSD.

**Table 1:** Quantitative results of evaluating the performance of the proposed multi-decoder segmentation network under individualistic and combined settings

DSC↑					NSD↑			
**Methods**	Individualistic (site 1)	Individualistic (site 2)	Individualistic (site 3)	**Average**	Individualistic (site 1)	Individualistic (site 2)	Individualistic (site 3)	**Average**
Individualistic (site 1)	78.24	67.66	65.1	70.33	77.23	65.4	63.7	68.78
Individualistic (site 2)	74.3	81.4	73.6	76.43	73.72	79.9	71.5	75.04
Individualistic (site 3)	75.45	71.3	80.9	75.88	73.12	72.41	81.13	75.55
Combined	79.1	80.1	78.8	79.33	76.7	78.5	79.6	78.27

In the individualistic scenario, we observed a significant degradation in generalization performance when the model was evaluated on test sets from other sites compared to the performance on the corresponding training data. For example, in the first row (corresponding site1-based training) the DSC is dropped from 78.24 to 67.66 when it comes to evaluation on site 2. Similarly, the NSD score dropped from 77.23 to 65.4 when it came to site 2. The behavior is notable for all individualistic scenarios, which confirms our claims about the impact of the distributional shift caused by muti-site medical data. To this end, we emphasize the importance of incorporating heterogeneous data during the training phase, which can be achieved through the combined learning approach offered by the multi-decoder segmentation network. In the combined training scenarios, we observed that the segmentation performance exhibited notable improvements compared to the individualistic scenario with unseen testing data. However, this remains below segmentation performance in the case of training and testing data. This suggests that leveraging data from multiple sites can enhance the model's ability to generalize across different data sources and improve overall segmentation performance.

To further evaluate the performance of the multi-decoder segmentation network, we conducted comparative experiments against state-of-the-art segmentation models that have demonstrated excellence in various medical imaging segmentation tasks. These models were carefully selected to provide a comprehensive evaluation and enable a meaningful comparison of our proposed approach. Table 2 presents the results of our comparative evaluation, where each model was trained and tested on the same dataset under consistent experimental conditions. These experiments aimed to assess and compare the performance of our proposed model against other existing models, providing valuable insights into the potential of our approach to advance the field of COVID-19 segmentation from multi-site data.

**Table 2:** Numerical comparison between the segmentation performance of the proposed multi-decoder segmentation network against the competing methods (mean ± standard deviation)

	DSC↑					**NSD↑**			
Models	Site 1	Site 2	Site 3	Total		Site 1	Site 2	Site 3	Total
U-Net [29]	82.1 ± 8.9	86.1 ± 9.9	87.1 ± 8.9	85.10 ± 9.23		80.9 ± 10.3	84.9 ± 11.1	87.1 ± 11.5	84.30 ± 10.97
FCN-8[44]	82.2 ± 9.1	86.2 ± 9.1	86.9 ± 10.1	85.10 ± 9.43		80.3 ± 11.2	84.4 ± 10.3	86.9 ± 10.3	83.87 ± 10.60
3D-U-Net [45]	80.8 ± 11.4	84.0 ± 8.4	85.9 ± 14.3	83.57 ± 11.37		79.7 ± 14.5	83.5 ± 9.8	85.2 ± 13.4	82.80 ± 12.57
3D-V-Net [26]	80.1 ± 11.2	84.7 ± 9.2	85.3 ± 15.1	83.37 ± 11.83		79.9 ± 13.1	84.6 ± 10.7	85.3 ± 13.3	83.27 ± 12.37
DSBN [12]	83.1 ± 6.8	86.9 ± 5.2	87.8 ± 6.9	85.93 ± 6.30		82.0 ± 8.3	85.7 ± 8.1	87.0 ± 6.5	84.90 ± 7.63
MS-Net [12]	83.8 ± 6.5	87.8 ± 6.4	88.5 ± 5.9	86.70 ± 6.27		82.9 ± 9.4	86.6 ± 8.7	87.9 ± 6.5	85.87 ± 8.20
Proposed	85.3 ± 5.3	89.2 ± 5.1	89.9 ± 6.1	88.13 ± 5.50		84.6 ± 7.1	88.2 ± 6.9	87.8 + 5.1	86.87 ± 6.37

Through these comparative experiments, we can demonstrate the efficacy and superiority of our proposed model. Our model consistently outperformed the other models in terms of various evaluation measures, showcasing its ability to achieve more accurate and reliable segmentation results. These findings highlight the effectiveness of our proposed multi-decoder segmentation network in handling the challenges posed by multi-site data in the context of COVID-19 segmentation. To further quantify the significance of the observed performance improvements, we conducted paired t-tests between the proposed multi-decoder segmentation network and the mixed and independent models using the evaluation measures. The statistical significance level was set at a p-value of 0.05. For each pair of comparisons, we calculated both the single source p-value and the total p-value. Table 3 displays the results of these statistical tests conducted on different datasets from various sites. Notably, all the computed p-values were found to be less than 0.05, indicating that the observed performance enhancements achieved by our proposed multi-decoder segmentation network are statistically significant. This signifies that the improvements observed in the segmentation results are not due to random chance, but rather reflect the true effectiveness and superiority of our model. These statistical tests provide further confidence in the reliability and robustness of our proposed multi-decoder segmentation network, reinforcing its potential for practical application in real-world scenarios. The statistically significant performance improvements observed across different datasets and sites validate the credibility and generalizability of our approach, making it a promising solution for accurate COVID-19 segmentation in the context of multi-site data.

**Table 3:** Statistical significance of the proposed against baseline

Method	Site 1	Site 2	Site 3
proposed vs U-Net [29]	7.84E-04	2.32E-11	3.95E-10
proposed vs FCN-8 [44]	1.07E-08	4.20E-09	3.92E-09
proposed vs DSBN [12]	4.47E-04	1.49E-02	2.16E-08
proposed vs MS-Net [12]	4.44E-07	4.00E-08	2.91E-03

### Ablation analysis

To gain insights into the impact of different feature recalibration blocks in the knowledge fusion from decoding paths in our model, we conducted an ablation analysis. We implemented and evaluated the KF module of our model with three variations of feature recalibration blocks: recombination and recalibration (RR), spatial SE (sSE), and a combination of channel-wise SE (cSE) and spatial SE (sSE). The results of these experiments are summarized in Table 4. The analysis reveals that the utilization of cSE blocks proves to be more effective than sSE blocks, emphasizing the importance of channel-wise information in the segmentation process. The cSE blocks enable the model to recalibrate the channel-wise features, enhancing their discriminative power and ultimately improving the segmentation performance. On the other hand, the RR blocks achieve comparable performance to the sSE blocks, suggesting that the recombination and recalibration mechanism successfully incorporates spatial information into the model. Furthermore, integrating both cSE and sSE blocks yields the highest performance across all evaluated datasets. This combination of feature recalibration mechanisms allows the Multi-decoder segmentation network to leverage both channel-wise and spatial information, leading to more accurate and robust segmentation results. However, it is worth noting that this integration comes at the cost of increased computational complexity, making it more computationally exhaustive compared to using individual recalibration blocks.

**Table 4:** ablation results for KF module in the proposed model

	**DSC↑**			**NSD↑**		
	**Site 1**	**Site 2**	**Site 3**	**Site 1**	**Site 2**	**Site 3**
RR [57]	84.4	88.5	88.9	88.03	84.06	88.0
sSE [37]	84.1	88.4	89.1	88.52	83.5	87.9
cSE [37]	85.3	89.2	89.9	88.13	84.6	88.2
cSE + sSE [37]	86.1	90.3	90.4	88.46	83.9	87.8

Considering the resource efficiency aspect, cSE blocks are shown to slightly increase the network parameters. However, the overall impact on computational resources remains relatively modest. This highlights the practicality and resource efficiency of integrating cSE blocks into the Multi-decoder segmentation network architecture, as they provide significant performance gains while maintaining manageable computational requirements. The ablation analysis on the feature recalibration blocks reaffirms the effectiveness and importance of channel-wise information in the context of the Multi-decoder segmentation network. It demonstrates that the integration of CSE blocks, in combination with other recalibration mechanisms, leads to superior segmentation performance. This analysis aids in the understanding of the design choices in our proposed model and supports the selection of CSE blocks as a key component in enabling effective interaction between decoding paths for enhanced COVID-19 segmentation.

### Scalability analysis

In this part of our experimental analysis, we conducted several experiments to investigate the relationship between the number of edges and the training accuracies.

**Fig. 4:**
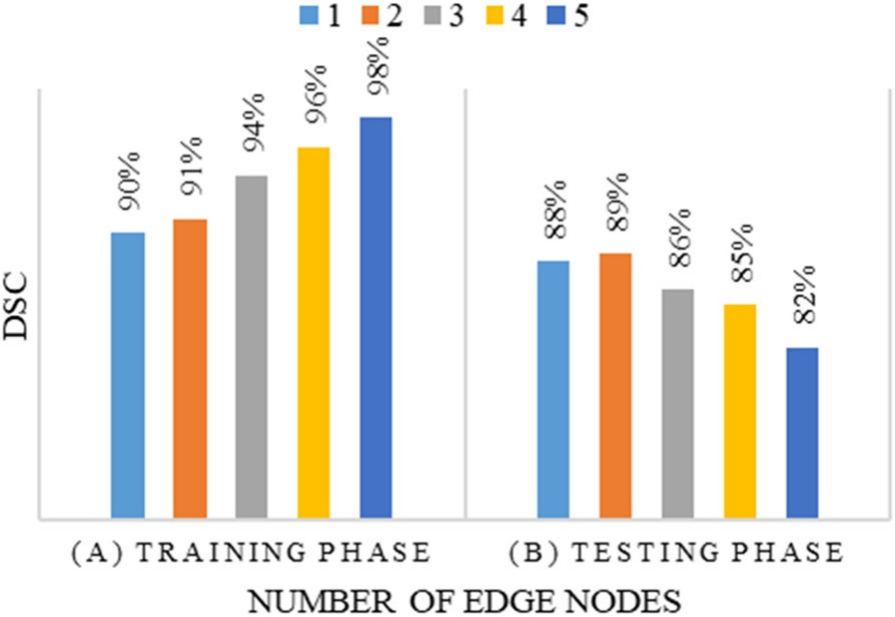
Impact of number of edges model accuracy

The results are presented in Fig. 4A, which illustrates how the training DSC varies with an increasing number of edge nodes. The findings reveal that as the number of edge nodes increases, the training DSC also increases. This can be attributed to the fact that when the number of nodes increases, the allocation of CT scans to each node decreases. Consequently, this reduction in the number of training samples per node leads to overfitting during training, resulting in higher training DSC scores. On the other hand, the impact of the number of nodes on the testing accuracy is depicted in Fig. 4B. The graph shows that a higher number of edges leads to a decrease in the testing DSC. This can be explained by the fact that with a larger number of edges, each edge node receives only a small portion of the training samples. As a result, the model trained on these limited samples may struggle to generalize well to unseen data, resulting in a decrease in testing accuracy. These observations highlight the importance of finding the right balance when determining the number of edges in the system. While a higher number of edges may lead to better training performance initially, it can also result in decreased testing accuracy due to limited training samples per edge node. Therefore, it is crucial to carefully consider the trade-off between training and testing performance when designing the system and selecting the appropriate number of edges.

### Time analysis

In consideration of real-world applications, we acknowledge the supreme standing of proficiency when it comes to being deployed in PANDFOG, for automatic COVID-19 pneumonia diagnosis. To this end, the feasibility and complexity of PANDFOG are assessed in clinical settings by conducting a set of time experiments to give useful insights about different time complexities.

**Fig. 5:**
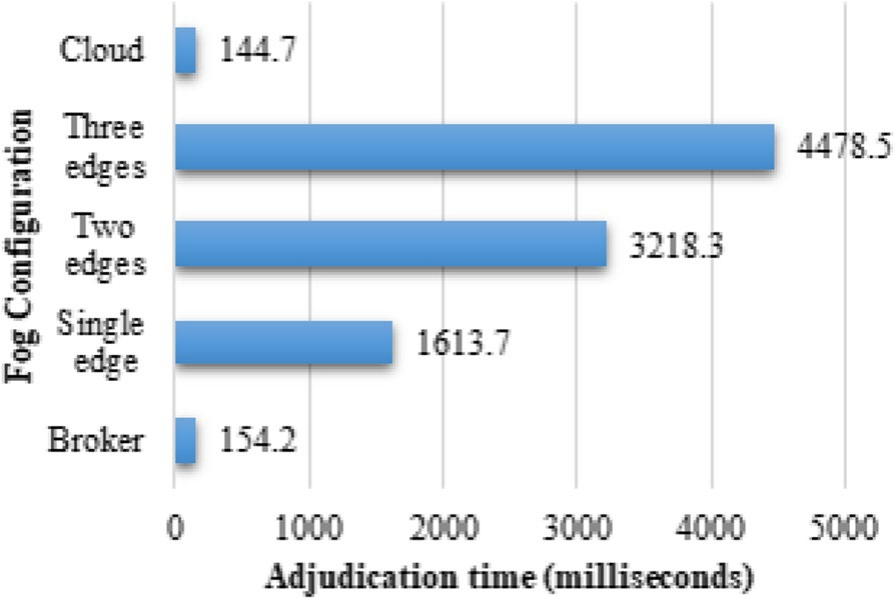
Adjudication time visualization in PANDFOG across various fog designs

In of adjudication time analysis, we conducted four experiments to evaluate the impact of adjudication time at the broker node for different fog paradigms. These paradigms included a cloud, a broker, a worker node, and dual worker nodes, as illustrated in Fig. 5. Our results showed that when the task was transferred to the master or cloud, the adjudication time was relatively small, approximately 144.7 ms and 154.2 ms, respectively. This indicates that the processing time was efficient when the task was handled by these centralized nodes. However, as the number of edge nodes increased, the broker became responsible for examining the workloads of each worker and selecting the worker with the lowest load to allocate the task. Consequently, the adjudication time increased due to the growing number of edge nodes that needed to be assessed by the broker. Furthermore, when the domain-specific data was directed to workers for heterogeneous learning, the broker no longer needed to execute load checks. This is because the selection of the mainstream class could be performed by any worker, eliminating the need for the broker's involvement in load distribution. These findings highlight the dynamic nature of the system and the influence of different fog paradigms on adjudication time. The increase in edge nodes introduces additional complexity and processing overhead, resulting in longer adjudication times. However, by allowing workers to handle domain-specific data for heterogeneous learning, the system becomes more efficient as the workload distribution is effectively delegated without requiring the broker's intervention in load checks.

**Fig. 6:**
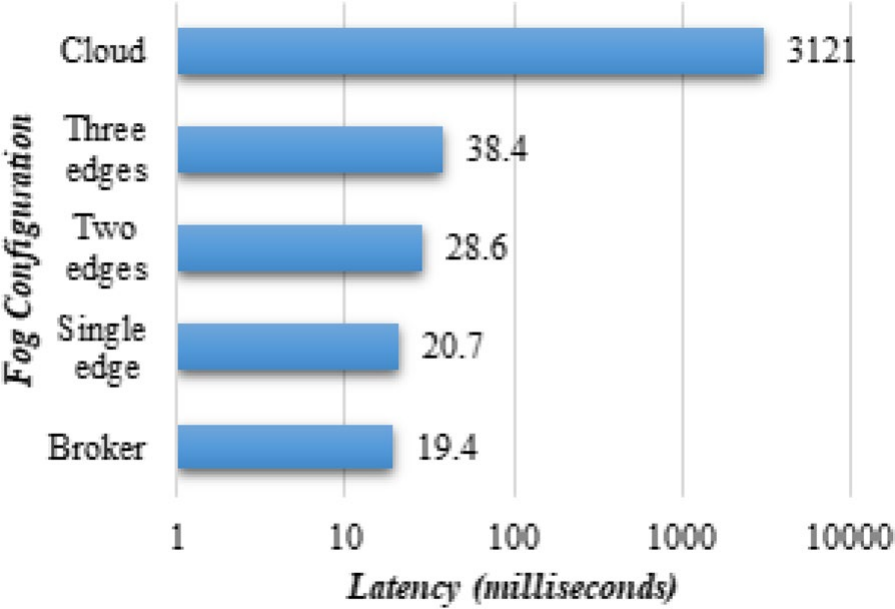
Comparative visualization of PANDFOG performance across different fog designs

In addition, we underline the significance of interpreting and evaluating the real-world applicability of PANDFOG for deploying the multi-decoder segmentation network on edge nodes. To this end, an extensive latency analysis is conducted to gain valuable feedback regarding the practical performance. Figure 6 illustrates the latency discrepancies, which include communication and queuing delays. It is observed that transmitting tasks to one edge or the broker node exhibits similar latency, approximately 19.4 ms and 20.7 ms, respectively, reflecting the total communication across one-hop data transmissions. In the multi-edge scenario, the latency slightly increases, reaching 28.6 ms for two edges and 38.4 ms for three edges. On the other hand, in the cloud scenario, a significantly higher latency of 3121 ms is observed due to the multi-hop transmissions of domain-relevant data out of the local area network (LAN). The achieved numerical results show the trade-offs and implications for data processing in smart cities, in which edge computing reduces latency for serious tasks, while multi-edge settings keep a balance between local processing and resource distribution. This, in turn, highlights the basics of optimizing network architecture to minimize latency and guarantee sensible decision support for managing pandemic disease within smart cities.

**Fig. 7:**
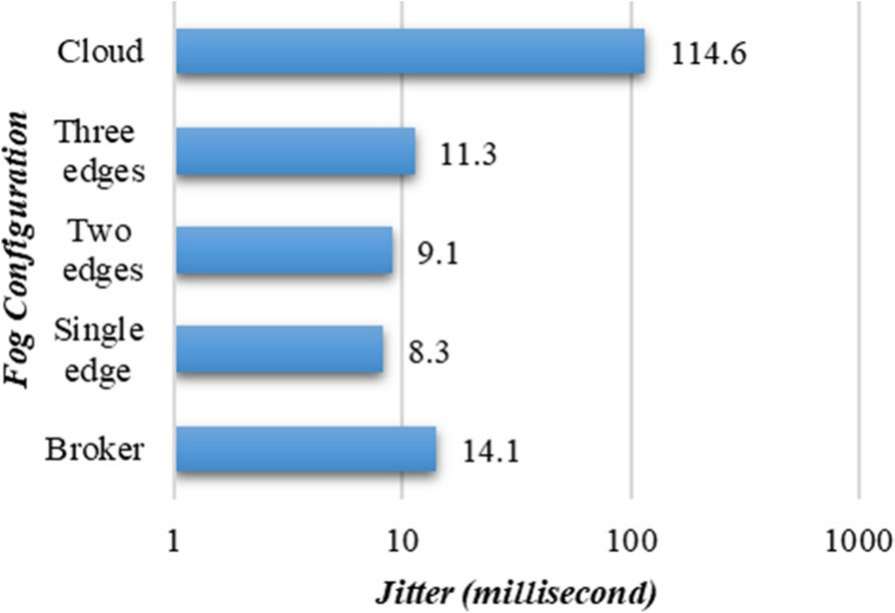
Jitter analysis of PANDFOG across varied fog designs

Moreover, the applicability of PANDFOG is further explored in real-world COVID-19 pneumonia analysis through the introduction of jitter analysis. This means studying the difference in response time of successive task requests, which is a significant parameter for real-time IoT services in smart cities. Figure. 7 depicts the jitter discrepancies along with different fog settings. The scenario involving only the broker node exhibits a higher jitter (14.1 ms) compared to the scenario where requests are directed to the worker nodes. This can be attributed to the additional adjudication induced by other tasks, while the broker is responsible for resource administration and security maintenance. Additionally, a marginal increase in jitter is observed for the three-edge scenario (13.1 ms) compared to the one-edge scenario (8.3 ms). Moreover, a larger jitter is observed when data is sent to a centralized cloud (114.6 ms).

**Fig. 8:**
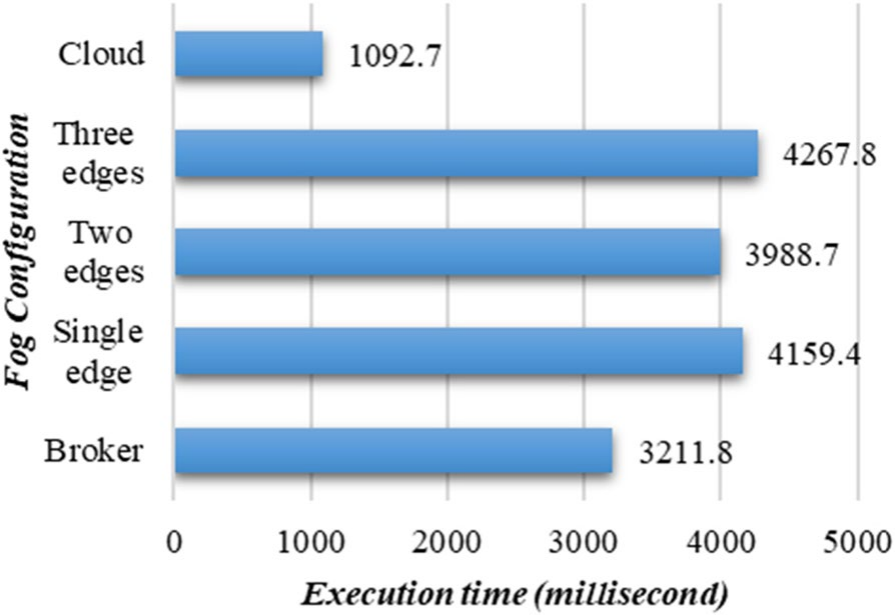
Comparative analysis of execution time in PANDFOG across various fog designs

Furthermore, Execution time analysis is broadly recognized as an essential metric for measuring the computing efficiency and responsiveness of a system when it comes to deployment into dynamic and constrained IoT systems, such as smart cities' healthcare infrastructure. The execution time analysis, as shown in Fig. 8, further highlights the differences in execution time across different scenarios. As expected, the cloud configuration demonstrates the lowest execution time (1092.7 ms) due to its high availability of computational resources. It is worth noting that the execution times of the worker nodes are considerably higher than those of the broker nodes.

### Bandwidth analysis

**Fig. 9:**
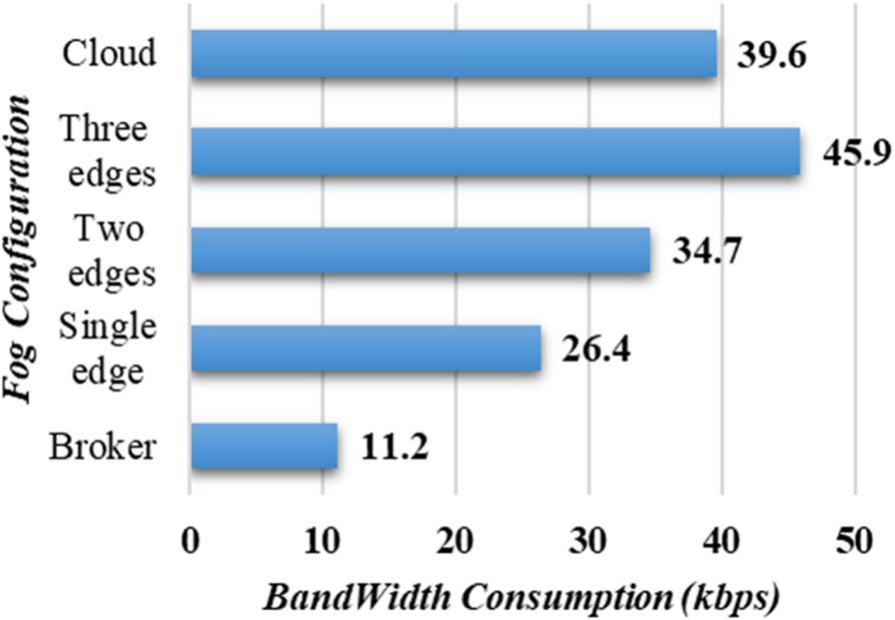
Bandwidth consumption analysis of PANDFOG with various fog design configurations

When it comes to analyzing distributed systems, such as smart city healthcare systems, bandwidth analysis comes as a critical metric for evaluating the viability and scalability of the system. To this end, network bandwidth analysis is performed for the proposed PANDFOG, a s displayed in Fig. 9, shows the relation between the network bandwidth consumption and the underlying fog configurations. The configuration involving only the broker node results in the lowest bandwidth consumption (11.2 kbps), while the cloud configuration exhibits comparatively higher consumption (39.6 kbps). Increasing the number of edge nodes leads to an increase in bandwidth consumption, primarily due to additional security checks, heartbeat packets, and data transmissions.

### Power analysis

To further get valuable insights into the real-world applicability of PANDFOG, the proposed

**Fig. 10:**
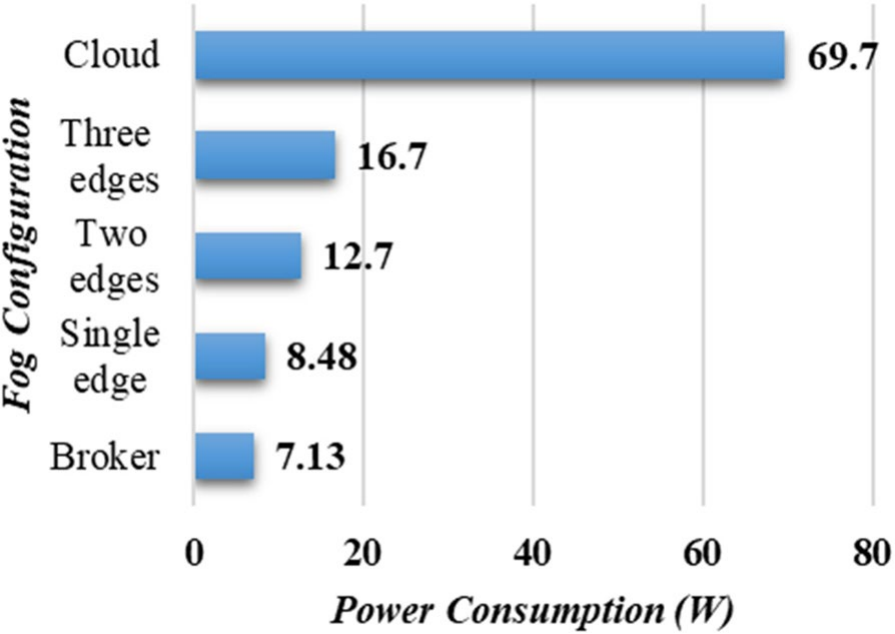
Power consumption analysis of PANDFOG under varied fog design configurations

Analyzing power and energy consumption, as depicted in Fig. 10, is a crucial aspect of IoT frameworks. The energy utilization analysis of the proposed COVID-19 fog framework indicates that the broker configuration has the lowest power utilization at 7.3 W. Additionally, it is observed that increasing the number of edge nodes only slightly increases power consumption. In contrast, the cloud configuration shows a significant increase in power consumption due to the abundance of computational resources it requires. These results demonstrate significant implications for the design and deployment of AIoT platforms in smart cities by emphasizing localized, edge-based data processing that not only reduces latency but also copes with energy competence and sustainability goals. The comparatively low power utilization in the case of broker configuration, fused with the medium spread at edge scaling, showcases the capacity for resource optimization in multi-edge deployments.

The above experimental analyses provide valuable insights into the performance, efficiency, and resource utilization of the proposed COVID-19 fog framework. The results highlight the trade-offs and characteristics of different configurations, helping to inform decision-making and optimization efforts in designing and deploying fog-based IoT systems for pandemic control in smart cities. Our research focuses primarily on the implication our work holds towards achieving the goal outlined under the Vision 2030 agenda particularly concerning developing futuristic smart cities. Our primary objective lies in developing a fog-based AIoT system for determining the best way in which, we could perform diagnosis for COVID-19-alike pandemics at the highest level of accuracy using novel approaches towards integrating multi-site data sourced from various multimedia scanners. This system allows smart cities to create an efficient system for tracking and monitoring pandemics, promoting public health safety and appropriate resource allocation. When implemented in existing healthcare facilities, our framework builds on existing medical wearables, electronic health records, and other related tools designed to holistically diagnose individuals while embracing personalized care aimed at promoting preventive measures. To summarize, our development of the cloud-based AIoT system will greatly aid in achieving the goals of Vision 2030 for intelligent cities. By providing precise detection and nurturing an interconnected healthcare infrastructure, our framework provides communities with the means to efficiently react to outbreaks and improve public safety, leading to greater quality of life for their inhabitants. Our accomplishment in implementing our setup creates opportunities for even larger changes within medicine and greases the wheels toward long-term, sustainable smart cities down the line.

## Conclusions and future works

This paper presents a comprehensive framework, named fog-based AIoT, for accurate pandemic disease diagnosis in smart cities. By leveraging multi-site data fusion, our framework addresses the challenges posed by heterogeneous data sources and enables precise and timely diagnosis of pandemic diseases, particularly focusing on COVID-19. The proposed AIoT framework incorporates fog-empowered cloud computing and integrates various hardware devices and software components to facilitate efficient data processing and analysis. The Multi-decoder segmentation network, our novel deep learning model, serves as the cornerstone of the framework, effectively handling the segmentation of COVID-19 lesions from CT scans obtained from diverse sources. The findings showed that our AIoT framework outperforms traditional centralized cloud systems in terms of precision, speed, and adaptability to heterogeneous data sources.

The successful functioning of our fog-based AIoT framework opens up new avenues for future research and applications in the field of disease diagnosis and monitoring in smart cities. Further progress can be made in optimizing resource allocation, refining the segmentation algorithms, and extending the framework to support other types of pandemic diseases. By constantly refining and expanding our AIoT framework, we can make significant strides towards creating smarter and more resilient cities that are better provided to tackle healthcare challenges during pandemics.

## Data Availability

The datasets generated during and/or analyzed during the current study are three publicly accessible datasets that are openly available in [40-42].

## References

[1] Elhosseini, M. A., Gharaibeh, N. K., & Abu-Ain, W. A. (2023). Trends in Smart Healthcare Systems for Smart Cities Applications. In 2023 1st International Conference on Advanced Innovations in Smart Cities (ICAISC) (pp. 1–6). IEEE.

[2] Singh PD, Kaur R, Dhiman G, Bojja GR (2023). BOSS: a new QoS aware blockchain assisted framework for secure and smart healthcare as a service. Expert Syst.

[3] Rejeb, A., Rejeb, K., Treiblmaier, H., Appolloni, A., Alghamdi, S., Alhasawi, Y., & Iranmanesh, M. (2023). The Internet of Things (IoT) in healthcare: Taking stock and moving forward. Internet of Things, 100721.

[4] Dang VA, Vu Khanh Q, Nguyen VH, Nguyen T, Nguyen DC (2023). Intelligent Healthcare: Integration of Emerging Technologies and Internet of Things for Humanity. Sensors.

[5] Bourechak A, Zedadra O, Kouahla MN, Guerrieri A, Seridi H, Fortino G (2023). At the Confluence of Artificial Intelligence and Edge Computing in IoT-Based Applications: A Review and New Perspectives. Sensors.

[6] Tripathy SS, Rath M, Tripathy N, Roy DS, Francis JSA, Bebortta S (2023). An Intelligent Health Care System in Fog Platform with Optimized Performance. Sustainability.

[7] Kumar M, Kumar A, Verma S, Bhattacharya P, Ghimire D, Kim SH, Hosen AS (2023). Healthcare Internet of Things (H-IoT): Current Trends, Future Prospects, Applications, Challenges, and Security Issues. Electronics.

[8] Rahman MA, Mahmud MT, Rahman MO (2020). IEF2C: A novel AI-powered framework for suspected COVID-19 patient detection and contact tracing in smart cities.

[9] Li E, Zeng L, Zhou Z, Chen X (2019). Edge AI: On-demand accelerating deep neural network inference via edge computing. IEEE Trans Wireless Commun.

[10] Bahbouh NM, Compte SS, Valdes JV, Sen AAA (2023). An empirical investigation into the altering health perspectives in the internet of health things. Int J Inf Technol.

[11] Kanellopoulos D, Sharma VK, Panagiotakopoulos T, Kameas A (2023). Networking Architectures and Protocols for IoT Applications in Smart Cities: Recent Developments and Perspectives. Electronics.

[12] Alenizi J, Alrashdi I (2023) SFMR-SH: Secure Framework for Mitigating Ransomware Attacks in Smart Healthcare Using Blockchain Technology. Sustain Machine Intell J. 2. 10.61185/SMIJ.2023.22104.

[13] Sallam, K., Mohamed, M. and Wagdy Mohamed , A. (2023) “Internet of Things (IoT) in Supply Chain Management: Challenges, Opportunities, and Best Practices”, *Sustainable Machine Intelligence Journal*, 2. 10.61185/SMIJ.2023.22103.

[14] Allam X, Z., & Jones, D. S. (2020). On the coronavirus (COVID-19) outbreak and the smart city network: universal data sharing standards coupled with artificial intelligence (AI) to benefit urban health monitoring and management. In Healthcare..

[15] Costa DG, Peixoto JPJ (2020). COVID-19 pandemic: a review of smart cities initiatives to face new outbreaks. IET Smart Cities.

[16] Shorfuzzaman M, Hossain MS, Alhamid MF (2021). Towards the sustainable development of smart cities through mass video surveillance: A response to the COVID-19 pandemic. Sustain Cities Soc.

[17] Ngabo D, Dong W, Ibeke E, Iwendi C, Masabo E (2021). Tackling pandemics in smart cities using machine learning architecture. Math Biosci Eng..

[18] Carmichael H, Coquet J, Sun R, Sang S, Groat D, Asch SM, Hernandez-Boussard T (2021). Learning from past respiratory failure patients to triage COVID-19 patient ventilator needs: A multi-institutional study. J Biomed Inform.

[19] Wismüller A, DSouza, A. M., Abidin, A. Z., Ali Vosoughi, M., Gange, C., Cortopassi, I. O., … & Bader, A. S.  (2022). Early-stage COVID-19 pandemic observations on pulmonary embolism using nationwide multi-institutional data harvesting. NPJ Digital Medicine.

[20] Hooper JE, Padera RF, Dolhnikoff M, da Silva LFF, Duarte-Neto AN, Kapp ME, Williamson AK (2021). A postmortem portrait of the coronavirus disease 2019 (COVID-19) pandemic: a large multi-institutional autopsy survey study. Arch Pathol Lab Med.

[21] Kaissis G, Ziller A, Passerat-Palmbach J, Ryffel T, Usynin D, Trask A, Braren R (2021). End-to-end privacy preserving deep learning on multi-institutional medical imaging. Nat Mach Intell.

[22] Herath HMKKMB, Karunasena GMKB, Herath HMWT (2021). Development of an IoT based systems to mitigate the impact of COVID-19 pandemic in smart cities. Machine intelligence and data analytics for sustainable future smart cities.

[23] Gade DS, Aithal PS (2021). Smart cities development during and post COVID-19 pandemic–a predictive analysis. Int J Manage Technol Soc Sci..

[24] Yang S, Chong Z (2021). Smart city projects against COVID-19: Quantitative evidence from China. Sustain Cities Soc.

[25] Umair M, Cheema MA, Cheema O, Li H, Lu H (2021). Impact of COVID-19 on IoT adoption in healthcare, smart homes, smart buildings, smart cities, transportation and industrial IoT. Sensors.

[26] Ma Z, Ma G, Miao Y, Liu X, Choo KKR, Yang R (2020). Lightweight Privacy-preserving Medical Diagnosis in Edge Computing. IEEE Trans Serv Comput.

[27] Ndiaye M, Oyewobi SS, Abu-Mahfouz AM, Hancke GP, Kurien AM, Djouani K (2020). IoT in the wake of COVID-19: A survey on contributions, challenges and evolution. Ieee Access.

[28] Ronneberger O, Fischer P, Brox T (2015). U-Net: Convolutional networks for biomedical image segmentation in *MICCAI*.

[29] Xie S, Girshick R, Dollár P, Tu Z, He K (2017). Aggregated residual transformations for deep neural networks. InProceedings of the IEEE conference on computer vision and pattern recognition.

[30] Ioffe S, Szegedy C. Batch normalization: Accelerating deep network training by reducing internal covariate shift. https://arxiv.org/abs/1502.03167. 2015.

[31] Chaudhar P, Choromanska A, Soatto S, LeCun Y, Baldassi C, Borgs C (2017). Entropy-sgd: Biasing gradient descent into wide valleys.” in International Conference on Learning Representations.

[32] Rundo L, Han C, Zhang J, Hataya R et al., “Cnn-based prostate zonal segmentation on t2-weighted mr images: A cross-dataset study,” in https://arxiv.org/abs/1903.12571. 2019.

[33] Hou S, Pan X, Loy CC, Wang Z, Lin D (2018). “Lifelong learning via progressive distillation and retrospection,” Comput. Vision-ECCV.

[34] Milletari F, Navab N, Ahmadi SA. “V-net: Fully convolutional neural networks for volumetric medical image segmentation,” in https://arxiv.org/abs/1606.04797. 2016.

[35] Gao Z, Chung J, Abdelrazek M, Leung S, Hau WK, Xian Z (2019). Privileged modality distillation for vessel border detection in intracoronary imaging. IEEE Trans Med Imaging.

[36] Roy AG, Navab N, Wachinger C (2018). Recalibrating fully convolutional networks with spatial and channel “squeeze and excitation” blocks. IEEE Trans Med Imaging.

[37] Tuli S, Mahmud R, Tuli S, Buyya R (2019). Fogbus: A blockchain-based lightweight framework for edge and fog computing. J Syst Softw.

[38] Ma J, Wang Y, An X, Ge X, Yu Z, Chen J, et al. "Towards Efficient COVID-19 CT Annotation: A Benchmark for Lung and Infection Segmentation," arXiv preprint arXiv:2004.12537. 2020.

[39] Morozov S, Andreychenko A, Pavlov N, Vladzymyrskyy A, Ledikhova N, Gombolevskiy V, et al. "MosMedData: Chest CT Scans With COVID-19 Related Findings Dataset," arXiv preprint arXiv:2005.06465 . 2020.

[40] MedSeg. Dataset: https://medicalsegmentation.com/covid19/

[41] S. Nikolov, S. Blackwell, R. Mendes, J. De Fauw, C. Meyer, C. Hughes*, et al.*, "Deep learning to achieve clinically applicable segmentation of head and neck anatomy for radiotherapy," *arXiv preprint *arXiv:1809.04430*,* 2018. 10.2196/26151 PMC8314151 34255661

[42] Zhang L, Wang X, Yang D, Sanford T, Harmon S, Turkbey B (2020). Generalizing deep learning for medical image segmentation to unseen domains via deep stacked transformation," *IEEE Transactions on Medical Imaging*.

[43] Long J, Shelhamer E, Darrell T (2015). Fully convolutional networks for semantic segmentation. in CVPR.

[44] Çiçek Ö, Abdulkadir A, Lienkamp SS, Brox T, Ronneberger O, "3D U-Net: Learning Dense Volumetric Segmentation from Sparse Annotation," *arXiv e-prints,* p. arXiv:1606.06650 . Available: https://ui.adsabs.harvard.edu/abs/2016arXiv160606650C

[45] Li X, Chen H, Qi X, Dou Q, Fu C-W, Heng P-A (2018). HDenseUNet: hybrid densely connected UNet for liver and tumor segmentation from CT volumes. IEEE Trans Med Imaging.

[46] Mohamed, M. (2023) “Empowering deep learning based organizational decision making: A Survey”, Sustain Machine Intell J. 3. 10.61185/SMIJ.2023.33105.

[47] Miao Y, Tong Q, Choo K-KR, Liu X, Deng RH, Li H (2019). Secure online/offline data sharing framework for cloud-assisted industrial internet of things. IEEE Inter Things J..

[48] Miao Y, Weng J, Liu X, Choo K-KR, Liu Z, Li H (2018). Enabling verifiable multiple keywords search over encrypted cloud data. Inf Sci.

[49] Ouyang T, Li R, Chen X, Zhou Z, Tang X (2019). Adaptive user-managed service placement for mobile edge computing: An online learning approach. in Proc. IEEE Conference on Computer Communications (INFOCOM’19).

[50] Dai P, Lui K, Xing H, Yu Z, Lee VC (2019). A learning algorithm for real-time service in vehicular networks with mobileedge computing. in Proc. IEEE International Conference on Communications (ICC’19).

[51] Wang F, C Zhang, Lui J, Zhu Y, Pang H, Sun L (2019). Intelligent edge assisted crowdcast with deep reinforcement learning for personalized qoe. in Proc. IEEE Conference on Computer Communications (INFOCOM’19).

[52] Lin CC, Deng DJ, Chih YL, Chiu HT (2019). Smart manufacturing scheduling with edge computing using multi-class deep q network, IEEE Transactions on Industrial Informatics.

[53] Naranjo PGV, Pooranian Z, Shojafar M, Conti M, Buyya R (2019). FOCAN: A Fog-supported smart city network architecture for management of applications in the Internet of Everything environments. Journal of Parallel and Distributed Computing.

[54] Baccarelli E, Naranjo PGV, Scarpiniti M, Shojafar M, Abawajy JH (2017). Fog of everything: Energy-efficient networked computing architectures, research challenges, and a case study. IEEE access.

[55] Ghosh S, Mukherjee A, Ghosh SK, Buyya R (2019). Mobi-IoST: mobility-aware cloud-fog-edge-iot collaborative framework for time-critical applications. IEEE Transact Net Sci Eng.

[56] Mahmud R, Koch FL, Buyya R (2018). Cloud-fog interoperability in IoT-enabled healthcare solutions. in Proceedings of the 19th international conference on distributed computing and networking.

[57] Pereira S, Pinto A, Amorim J, Ribeiro A, Alves V, Silva CA (2019). Adaptive feature recombination and recalibration for semantic segmentation with fully convolutional networks. IEEE Trans Med Imaging.

